# The congenital APOA1 K107del mutation disrupts the lipid-free conformation of monomeric APOA1 and impairs oligomerization

**DOI:** 10.1016/j.jlr.2025.100929

**Published:** 2025-10-29

**Authors:** Ivo Díaz Ludovico, Marina C. Gonzalez, Horacio A. Garda, Romina F. Vázquez, Sabina Maté, María A. Tricerri, Nahuel A. Ramella, Shimpi Bedi, Jamie Morris, Scott E. Street, Esmond Geh, Geremy C. Clair, W. Sean Davidson, John T. Melchior

**Affiliations:** 1 Biological Sciences Division, Pacific Northwest National Laboratory, Richland, WA; 2 Instituto de Investigaciones Bioquímicas de La Plata “Prof. Dr. Rodolfo R. Brenner” (INIBIOLP), CONICET, CCT-La Plata, La Plata, Buenos Aires, Argentina; 3 Facultad de Ciencias Médicas, Universidad Nacional de La Plata (UNLP), La Plata, Buenos Aires, Argentina; 4 Department of Pathology and Laboratory Medicine, University of Cincinnati College of Medicine, Cincinnati; 5 Department of Neurology, Oregon Health and Science University, Portland, OR

**Keywords:** APOA1 self-association, lipid-free APOA1, APOA1 oligomerization, APOA1 (p.K131del), protein lipid interaction, cross-linking LC-MS, high-density lipoprotein (HDL)

## Abstract

Apolipoprotein A-I (APOA1) oligomerization is thought to be essential for high-density lipoprotein (HDL) formation and metabolism. Naturally occurring mutations can disrupt normal APOA1 folding and self-association, leading to dysfunctional HDL formation and cardiovascular disease. The congenital APOA1 variant p.K131del (APOA1^K107del^) has been associated with cardiovascular pathologies such as low HDL-cholesterol levels and aortic amyloidosis, and multiple studies indicate structural changes in APOA1 conformation underlie associated dysfunction. In the current study, we confirmed that APOA1^K107del^ exhibits no notable defect in lipid-binding. However, using polyacrylamide gel electrophoresis (PAGE) and size-exclusion chromatography (SEC), we found that loss of lysine 107 resulted in a remarkable shift in the distribution of APOA1 oligomers with a much higher proportion of monomers present in APOA1^K107del^ compared to wild-type APOA1. Further investigation using quantitative cross-linking revealed a major disruption of interactions in helical regions reported to participate in domain swaps necessary for proper self-association. This structural disruption appears to impair N- and C-termini interactions and dynamics that lead to non-specific aggregation. These findings support the hypothesis that lysine 107 is critical for proper folding and self-association of lipid-free APOA1 which could impact HDL biogenesis.

Apolipoprotein A-I (APOA1) is the primary protein component of high-density lipoproteins (HDL), where it plays a critical role in modulating lipid metabolism and cardiovascular health. It is best known for its role in the reverse cholesterol transport (RCT) pathway, which facilitates the transfer of cholesterol from peripheral tissues back to the liver for excretion, thereby exerting protective effects against the development of atherosclerotic lesions in the artery wall. However, we now know HDL has pleiotropic functionality participating in several key physiological processes to modulate whole-body metabolism. APOA1 plays a direct role in regulating many of these processes through interactions with lipids, other proteins, and cell surface receptors that exist in the body.

To date, more than fifty *APOA1* gene variants have been identified, with several linked to defective HDL metabolism (1, 2, 3, 4, 5, 6). Among these mutations is the congenital absence of lysine 107, p.K131del (APOA1^K107del^) (7), which remains the most commonly documented *APOA1* variant in Germany (8). This mutation is associated with low levels of circulating HDL cholesterol (9, 10, 11), hypertriglyceridemia (8, 12), ischemic heart disease (10, 13), and aortic amyloidosis (9, 13, 14). However, studies investigating the impact of deletion of K107 on processes important for HDL formation such as its efficiency to efflux cholesterol (15, 16), capacity to bind and solubilize HDL-sized micelles and liposomes (12, 16, 17, 18, 19, 20), and ability to bind and activate lecithin:cholesterol acyltransferase (LCAT) on nascent discoidal HDL (10, 21, 22) have yielded mixed results. Recent structural work indicates that deletion of K107 from APOA1 enhances binding to triglyceride-rich lipoproteins (TRLs) (17) such as very-low density lipoproteins (VLDL) and chylomicrons. Further, authors have shown that this association appears to impair lipolysis, possibly explaining the hypertriglyceridemia observed in these individuals. However, the structural underpinnings that explain this increased affinity are not well understood.

An intrinsic feature of lipid-free APOA1 is its ability to self-associate, which occurs at concentrations ≥0.1 mg/ml. A crystal structure of dimeric APOA1 (23), missing the C-terminus, revealed that, similar to lipid-free APOA4 (24, 25), helical domains of APOA1 participate in a domain swap when they self-associate. Domain swapping refers to a process in which two or more identical protein molecules exchange specific structural segments, resulting in the formation of interlinked dimers or higher-order oligomers. Remarkably, this crystal structure nicely recapitulated the “double belt” configuration of APOA1 that occurs on nascent HDL discs (26), indicating arrangements in the lipid-free state play an important role in interaction with ATP-binding cassette transporter A1 (ABCA1) for initial HDL biogenesis. Proper APOA1 configuration on the HDL surface is critical for binding and activation of critical modifying enzymes such as LCAT (27), directly linked to HDL deficiencies and development of cardiovascular disease (4), underscoring the importance of APOA1 structure as a key driver of HDL function.

In a previous study, we observed a reduced tendency of lipid-free APOA1^K107del^ to dimerize (19). In the current study, we follow up on these observations by more deeply investigating how loss of K107 impacts the ability of APOA1 molecules to interact in the lipid-free state. Using a combination of high-resolution size exclusion chromatography, chemical cross-linking and mass spectrometry (28), we characterized the solution structures of monomeric, dimeric, trimeric and tetrameric APOA1^K107del^. Our findings indicate that deletion of K107 leads to a significant structural disruption in the helical domain of APOA1 that is critical for proper self-interaction. Herein, we present our findings and discuss the impact of these structural changes on APOA1 function, lipoprotein assembly, and resulting defective HDL metabolism observed in carriers of the APOA1^K107del^ mutation.

## Materials and methods

### Protein expression and purification

Recombinant proteins were expressed and purified as previously described (18, 19, 29). Briefly, the cDNA of APOA1 with the wild type sequence (APOA1) and the mutant having a single deletion of the residue lysine 107 (APOA1^K107del^), were modified to introduce an acid-labile Asp-Pro peptide bond between amino acid residues 2 and 3, which allowed specific chemical cleavage of an N-terminal His-Tag fusion peptide. The constructs were inserted into a pET-30 plasmid (Novagen, Madison, WI) and transformed into BL21 (DE) *Escherichia coli* cells. Cells were grown in Luria-Bertani (LB) medium in the presence of kanamycin at 50 μg/ml at 37°C until the absorbance at 600 nm reached 0.5 arbitrary units. Protein expression was induced by the addition of 0.4 mM isopropyl β-d-thiogalactopyranoside (IPTG), followed by shaking at 140 RPM for 16 h at 28°C. Bacteria were spun and the pellet resuspended in equilibration buffer (EQB) NaH_2_PO_4_ 25 mM; NaCl 500 mM; 0,05% m/v of sodium azide, pH 8.0, plus 3 M guanidine hydrochloride, stirred overnight at 4°C. The sample was sonicated in an ice bath to further lyse cells, and insoluble debris was pelleted through a second centrifugation step. The supernatant was collected and dialyzed extensively against EQB and applied to immobilized metal chelate affinity chromatography (IMAC) Ni^2+^chelating columns (Novagen, Madison, WI), and purified as previously described (29, 30). His-tagged protein was eluted, collected, and the His-tag removed by incubation with 50% formic acid (v/v) for 5 h at 55 °C (29, 30). To purify the proteins away from the cleaved tag, the sample was reapplied to the IMAC) Ni^2+^-chelating columns and flow-through with pure protein collected and dialyzed against 10 mM Tris, pH 7.4. Final protein purity was at least 95% as determined by 16% SDS-PAGE stained with Coomassie Blue dye. Pure proteins were dialyzed into 10 mM Tris pH 7.4, lyophilized and stored at −80°C. When ready for studies, proteins were resolubilized in Tris buffer containing 3 M guanidine HCl at 1 mg/ml for 2 h at 4°C and then exhaustively dialyzed into the appropriate buffer for experimentation. Protein concentration was calculated by absorbance at 280 nm (31).

### Surface pressure measurements on Langmuir monolayers

Surface-pressure experiments were carried out with a KSV NIMA Langmuir-trough Model 102M (KSV-NIMA Biolin Scientific) with a Wilhelmy KN 0005 (or Whatman CHR1) filter paper (prewashed) as the surface-pressure sensor. The aqueous subphase consisted of phosphate-buffered saline (PBS) containing 137 mM NaCl, 2.7 mM KCl, 10 mM Na_2_HPO_4_, and 1.8 mM KH_2_PO_4_, pH 7.4. The 1-palmitoyl-2-oleoyl-sn-glycero-3-phosphocholine (POPC) or lipid mixture POPC/Cholesterol at 4:1 M ratio dissolved in chloroform was gently spread over the surface of a Teflon custom-built micro trough containing 6.5 ml of the subphase until the desired initial surface pressure (Πo) was attained without barrier compression. APOA1 or APOA1^K107del^ were then injected into the subphase bulk with a beveled-tip Hamilton syringe to a 1.5 μg/ml final concentration. The increment in surface pressure (ΔΠ) versus time was recorded until a stable signal was obtained. All the experiments were carried out at 26 ± 1 °C. The exclusion surface pressures were determined by fitting the ΔΠ versus Πo data to a linear equation (y = mx + b) and extrapolating to y = 0. Exclusion surface pressure values were compared using a *t* test.

### Measurement of Intrinsic fluorescence of tryptophan

Fluorescence emission spectra were acquired in a Photon Technology International Quantamaster spectrometer in photon counting mode, with excitation at 295 nm and emission registered between 300 and 400 nm in PBS buffer. The Raman scattering contribution was obtained from spectra of PBS alone within the same range and subtracted from sample spectra to detect fluorescence maxima.

### Cross-linking of proteins and oligomers isolation

Proteins were brought to 1 mg/ml in PBS and cross-linked using Bis(sulfosuccinimidyl)suberate (BS^3^) at a molar ratio of 50:1 BS^3^:APOA1 for 12 h at 4°C (28). The reaction was quenched by bringing the final concentration of the sample to 50 mM Tris-HCl and incubating for 15 min at room temperature. For isolation of the species, we concentrated a total of 4 mg of cross-linked samples to 250 μl using 10,000 kDa MWCO Amicon® concentrators (Millipore and applied the samples to three Superdex 200 gel filtration columns (10/300 Gl; GE Healthcare) hooked in tandem as previously described (28). Species were eluted in PBS at a flow rate of 0.3 ml/min into 0.5 ml fractions. Purity of the fractions for each species was determined using a 4%–15% SDS-PAGE stained with Coomassie blue. Fractions containing high purity of each respective cross-linked species were pooled, protein concentration was determined by Markwell Lowry protein assay (32), and samples were stored at 4 °C until ready for further analysis.

### Analysis of cross-linked oligomers using mass spectrometry

Cross-linked samples were extensively dialyzed against 50 mM NH_4_HCO_3_, pH 8.1 at 4°C. A total of 100 μg of each oligomeric species of APOA1 and APOA1^K107del^ were subsequently digested using sequencing-grade trypsin (Promega) at a final ratio of 1:20 (protein/trypsin) at 37°C for 16 h. Samples were then taken to dryness and stored at −20°C. For mass spec analysis, the peptides were solubilized in 15 μl of 0.1% (vol/vol) formic acid, vortexed, and incubated at RT for 10 min. Samples were centrifuged at 10,000 x g for 10 min to remove any impurities and 10 μl of supernatant was transferred to an MS sample vial. A total of 8 μg of each sample was applied to the C18 IntegraFrit trap column. Peptides were eluted at flow rate of 0.1 ml/min. The mobile phase consisted of solvent A (0.1% formic acid in water) and solvent B (0.1% formic acid in acetonitrile). The gradient was programmed as follows: mobile phase gradient from 95% A for 5 min, decreased to 68% A at 120 min, 50% A at 122 min, and 10% A at 123 min, followed by re-equilibration to 95% A at 134 min. The eluted peptides were introduced into the mass spectrometer using a Dual Agilent Jet Stream (AJS) electrospray ionization (ESI) source, operating in positive ion mode. The iFunnel Q-TOF Agilent 6550 instrument was operated to acquire data using the following settings: full MS scans were collected over an *m/z* range of 300–1400 at an acquisition rate of 5 spectra per second (200 ms per spectrum), while MS/MS scans were acquired over an *m/z* range of 100–1700 at a rate of 3 spectra per second (333 ms per spectrum). Collision energy (CE) was applied dynamically according to precursor charge state using the following formulas: for triply charged precursors (z = 3), CE was calculated as CE = 4 × (m/z)/100 − 2; for precursors with charge greater than 3 (z > 3).

### Identification of cross-linked peptides

Initial cross-links were identified using the Spectrum Identification Machine for cross-linked Peptides software (SIM-XL) version 1.5.4.0. Search parameters that were used included setting disuccinimidyl Suberate (DSS) as the crosslinker, which has identical mass shift modifications as BS^3^. Searches were performed, including oxidation of methionine and formylation as variable modifications. The fragmentation method used was collision-induced dissociation (CID). Identifications allowed for up to 3 missed cleavages, and searches were performed between 300 to 3,000 MH with a tolerance of 2.0 s (28, 33). As a control, we also searched for mass spec data obtained on non cross-linked APOA1 and APOA1^K107del^. Each identified cross-link was manually verified through visualization of MS1 spectra and only cross-linked peptides having differences <∣0.05∣ between theoretical and experimental M + H were considered for further analysis. We next used MassHunter Workstation Quantitative Analysis software version B.07.00 to manually identify MS1 peaks of bona-fide cross-links and quantify the area under the curve (AUC) with the baseline manually determined for each curve for its corresponding m/z value.

### Quantification of cross-linked peptide abundance

To ensure an equal mass of cross-linked peptides was injected into the mass spectrometer, we normalized the data to a common peptide in each species lacking a lysine, i.e. non-modified. To identify the peptide candidates, we searched the cross-linked data files for both APOA1 and APOA1^K107del^ using MASCOT. Methionine oxidization was set as a variable modification, and trypsin as enzyme for digestion. The peptide (R)THLAPYSDELR(Q) MH+3 = 434.5500 was selected for normalization as it was in high abundance and present in all samples. Normalization was performed by dividing the AUC of the cross-linked peptides within each sample by the AUC of the non-modified peptide in the same sample. In the case that cross-links were determined between the same lysines, but peptide masses were different due to missed cleavages, oxidation, or presence of a hydrolyzed cross-linker, we summed the AUCs together for that particular cross-link.

### Statistical Analysis

Statistical significance between APOA1 and APOA1^K107del^ cross-linked peptides was determined by unpaired t-tests. Each comparison was analyzed individually, assuming Gaussian distribution, with no assumption about consistent standard deviation (Welch correction) in the software GraphPad Prism version 10.1.3. The uniqueness of a cross-link was defined as not being detectable in the three replicates of APOA1 or APOA1^K107del^ and being quantifiable by AUC in the other three replicates.

## Results

### APOA1^K107del^ exhibits no defect in its capacity to interact with lipids

**Fig. 1:**
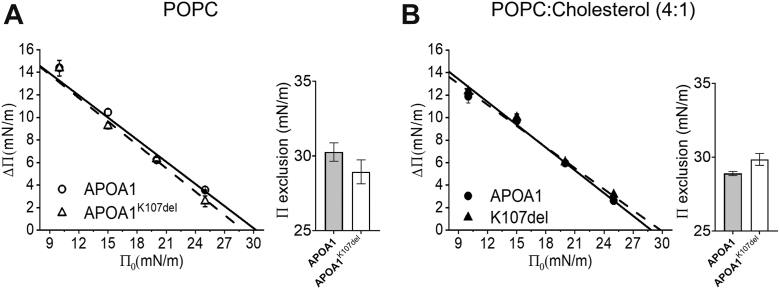
APOA1 and APOA1^K107del^ interaction with lipid monolayers. Adsorption of APOA1 or APOA1^K107del^ to lipid interfaces of 1-palmitoyl-2-oleoyl-sn-glycero-3-phosphocholine (POPC) in panel A and POPC:Cholesterol (4:1) in panel B was registered as changes in surface pressure ΔΠ in the y-axis as a function of initial surface pressure Π_0_, x-axis. Bar graphs show the exclusion surface pressures for each lipid monolayer composition composition as the mean ± standard error of two independent experiments.

In a previous study, we reported that the K107 deletion had no impact on lipid interaction using small unilamellar vesicles composed of POPC or POPC:Cholesterol (19). To explore whether lipid curvature could influence this interaction, we evaluated the ability of WT APOA1 and APOA1^K107del^ to adsorb to planar lipid monolayers. Using the same lipid compositions in surface balance studies, we observed that both APOA1 and APOA1^K107del^ adsorbed to POPC monolayers in nearly identical ways (Fig. 1A), and the addition of cholesterol had no measurable effect on protein adsorption (Fig. 1B). Exclusion pressure was determined by extrapolating the fitted isotherms, with values calculated at y = 0. For POPC alone, exclusion pressures were 30.3 ± 0.7 mN/m for WT APOA1 and 28.9 ± 0.8 mN/m for APOA1^K107del^. In the presence of cholesterol, values were 28.9 ± 0.1 mN/m and 29.8 ± 0.4 mN/m for WT and mutant, respectively. *t* test analysis revealed no significant differences in either exclusion pressure or isotherms slope across all conditions tested. These findings are consistent with our previous liposome studies and support the notion that lysine 107 is not essential for APOA1–lipid interaction (18, 19, 20).

### Lysine 107 modulates APOA1 self-association

**Fig. 2:**
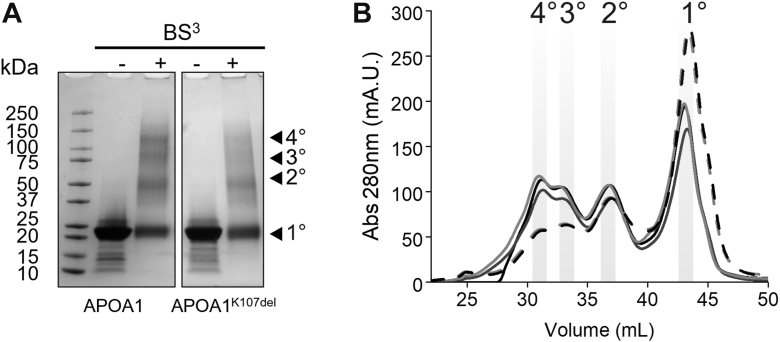
Crosslinking of lipid-free APOA1 and APOA1^K107del^. Proteins were cross-linked with BS^3^ and analyzed by SDS-PAGE and size exclusion chromatography. A: SDS-PAGE 4%–15% gradient gel of APOA1 and APOA1^k107del^ before (−) and after (+) cross-linking. Oligomeric states are denoted as monomers (1°), dimers (2°), trimers (3°) and tetramers (4°). Each lane contains 8 μg of protein which was visualized using Coomassie blue stain. B: Cross-linked samples were analyzed by size-exclusion chromatography using three Superdex 200 columns in series. Protein was monitored as absorbance at 280 nm. The solid line represents APOA1 and dashed lines represent APOA1^K107del^. Three replicates of each protein were analyzed. Shaded areas represent fractions collected of each oligomer species for downstream analysis.

We previously reported a reduced capacity of APOA1^K107del^ to form dimers (19). To determine whether this was due to disrupted self-association or an artifact of the crosslinking method, we compared the self-association patterns of WT APOA1 and APOA1^K107del^ using native-PAGE. WT APOA1 is known to self-associate at concentrations as low as 0.1 mg/ml (28) so we tested a range of concentrations from 0.05 mg/ml to 1.0 mg/ml of each protein. Native-PAGE analysis revealed a slight decline in the high molecular weight range of APOA1^K107del^ at all concentrations above 0.1 mg/ml (Supplementary Fig. 1A) supporting the notion that deletion of lysine 107 disrupts the ability of APOA1 to self-associate. To gain more resolution in these interactions, we used chemical cross-linking to “lock” the different oligomers in place. Proteins were cross-linked at 1 mg/ml and SDS-PAGE analysis confirmed a reduction in oligomer formation in APOA1^K107del^ (Fig. 2A, Supplementary Fig. 1B) compared to WT APOA1. To further characterize these species, we turned to high-resolution size exclusion chromatography to isolate monomers, dimers, trimers and tetramers of each protein (28). Equal masses of cross-linked samples were applied to the columns and we observed striking differences in the elution pattern (Fig. 2B). Cross-linked APOA1^K107del^ had increases in monomeric species and concomitant decreases in dimers, trimers and tetramers compared to WT APOA1. Fractions were tested using SDS-PAGE to pool those with the highest purity within each species for downstream structural analysis (Supplementary Fig. 1C and D).

### Deletion of lysine 107 disrupts APOA1 tertiary and quaternary structure

Next, we investigated the structural impact due to the deletion of lysine 107. First, we turned to intrinsic fluorescence to monitor changes in the exposure to solvent of tryptophan. We previously used this technique to demonstrate a shift in fluorescence in APOA1^K107del^ compared to WT APOA1 (19). Here, we tested each cross-linked species and observed a ∼2 nm red shift in the spectral maxima of APOA1^K107del^ for all species indicating the structural alteration was not unique to a specific quaternary assembly, but that it exists across all self-associated structures (Supplementary Fig. 2). These data indicate more exposure of tryptophan residues suggesting that the structure may be less compact and more intrinsically disordered in nature.

### Deletion of lysine 107 destabilizes APOA1 N- and C-termini

**Fig. 3:**
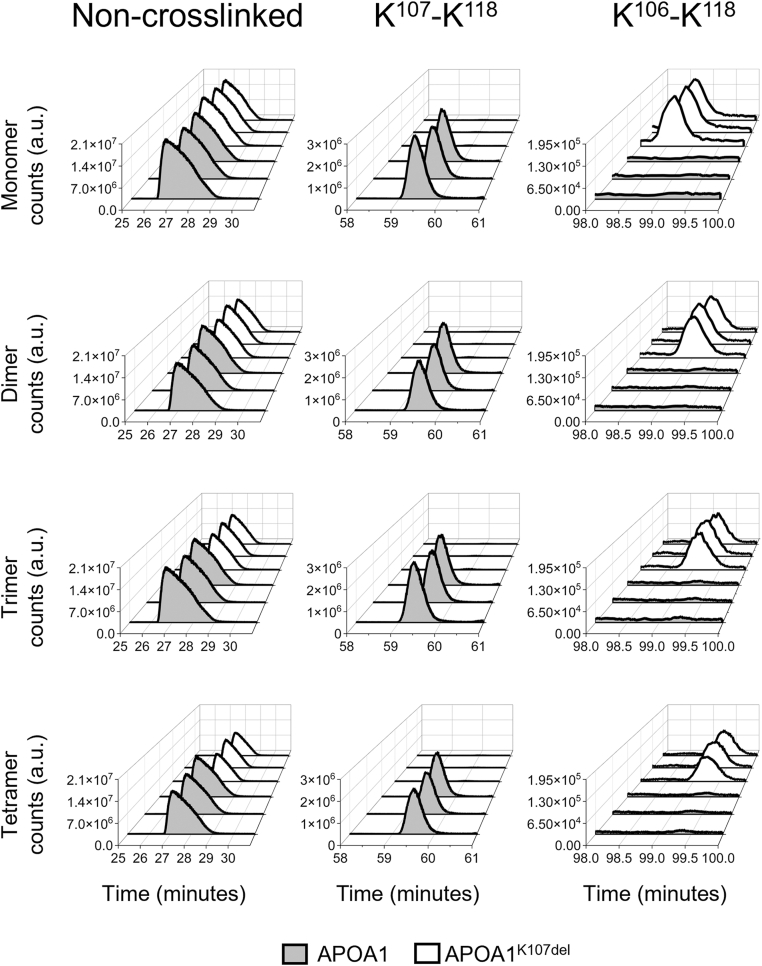
Extracted ion chromatograms (EIC) of controls and crosslinked peptides in APOA1 and APOA1^K107del^. The left column shows the EIC of the non-modified peptide ^59^THLAPYSDELR^169^ with m/z = 434.55 that was used for normalization. The middle column shows the EIC of ^107^KWQEEMELYRQKVEPLR^123^ containing an intrapeptide cross-link between K^107^- K^118^ with m/z = 605.32. The right column shows the EIC of ^95^AKVQPYLDDFQKWQEEMELYRQKVEPLR^123^ containing an intrapeptide cross-link between K^106^ - K^118^ with m/z = 970.50.

Next, we performed a more detailed analysis by examining changes in the cross-linking patterns between WT APOA1 and APOA1^K107del^ for all the species. Given that APOA1^K107del^ still forms some level of oligomers, we developed an approach to quantify differences in abundance of the cross-links within species in both WT and APOA1^K107del^. This required that we ensure that an equal mass of the sample is analyzed by the mass spectrometer. To account for potential subtle changes in the injection mass, we normalized our data using the non-modified peptide ^159^THLAPYSDELR^169^. This peptide lacks lysines, meaning it can not be modified by BS^3^ and further lacks methionines, which can be oxidized and skew analysis. To quantify peptide or cross-link abundance, we extracted parent ion masses and measured the area under the curve. The abundance of the non-modified peptide across each species is shown in the left column in Fig. 3. Notably, we show two unique cross-links involving K118, the first one to K107 found only in the WT and the second one to K106 found only in APOA1^K107del^ confirming the absence of K107 in the mutant protein. In total, we identified 32 different cross-links across the different species. A comprehensive list of all the cross-linked species and their relative abundance is provided in Supplemental Table 1.

**Fig. 4:**
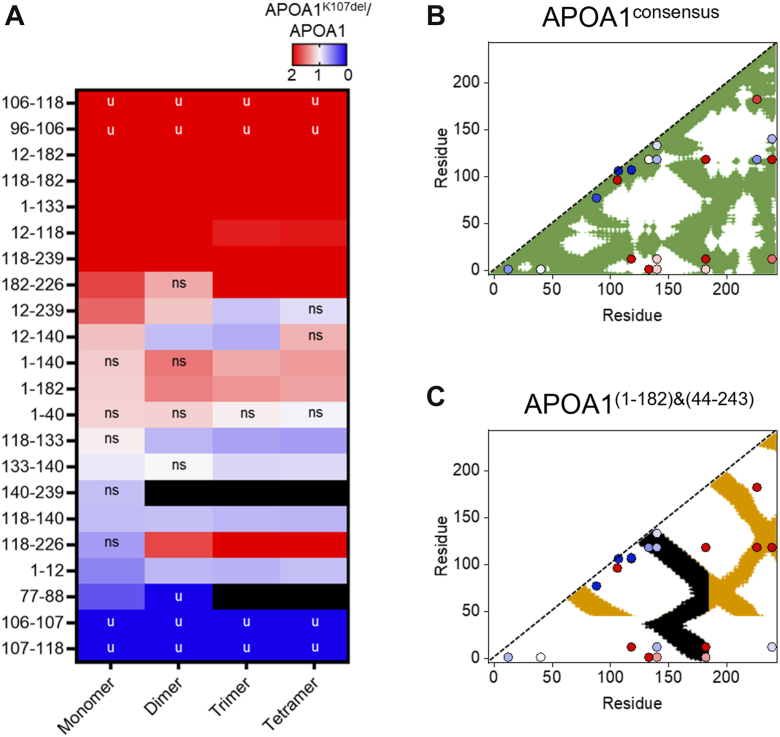
Changes in cross-linking abundance in APOA1^K107del^ oligomers. Relative changes in abundance of cross-links in APOA1^K107del^ compared to APOA1 controls. A: Heatmap of changes in cross-link abundance expressed as a ratio of APOA1^K107del^: APOA1 for each biophysical state. Cross-linked residues are displayed on the y-axis. All cross-links were statistically significant unless marked as non-significant (ns). Unique cross-links, only found in each variant are marked with “u”. Crosslinks present in one oligomeric state but not another are represented by black filling. Values represent three replicates of each cross-link. Statistical significance was determined using Welch’s *t* test. B: Changes in cross-link abundance in monomeric APOA1K107del superimposed on a contact plot generated for the consensus APOA1 structure (34). C: Changes in cross-link abundance median across dimers, trimers, and tetramers of APOA1^K107del^ superimposed on a contact plot generated for intermolecular contact plots from a crystal structure of the C-terminally truncated dimer (black, (23)) and the N-terminally truncated tetramer (yellow, (35)). Contact plots were generated with an upper limit of 28 Å. Cross-links are color-coded by abundance changes shown in panel A.

A comparative analysis contrasting changes in protein abundance across the different species is displayed as a heatmap in Fig. 4A. Cross-links that increased in abundance in the APOA1^K107del^ species are shown in red while those decreased are shown in blue. We identified four unique cross-links that only showed up in either the WT APOA1 or APOA1^K107del^ cross-linked species. These were primarily limited to cross-links involving K106 or K107 with K106 exclusive to the mutant and K107 exclusive to WT APOA1. Similar to the intrinsic fluorescence analysis, changes in the patterns of cross-linking appear to persist across all oligomeric states. However, a few exceptions were observed. For instance, the K12-K140 cross-link increased in APOA1^K107del^ monomers but decreased in its dimers and trimers. Similarly, the K77-K88 cross-link was reduced threefold in APOA1^K107del^ monomers, was present only in APOA1 dimers, and was undetectable in higher-order oligomers of both APOA1 and APOA1^K107del^.

Finally, we aimed to determine whether changes in these cross-linking patterns appear specific to any key domains on APOA1. To investigate this, we first turned to the consensus structure of lipid-free monomeric APOA1 (34) and the crystal structures (23, 35) of self-associated APOA1. First, we generated a contact plot that provides proximity information on Cα distance within the structures using PROTMAP2 v1.2.2. The consensus model is monomeric and thus the map displays, by definition, intramolecular distances (Fig. 4B). The crystal structures have both intra- and inter-molecular cross-links. We were only interested in investigating how cross-links overlayed with the inter-molecular distances so only those were included in the map for simplicity (Fig. 4C). We then overlayed the cross-linked residues with colors representing their heat index changes in abundance.

The contact plots revealed that deletion of K107 resulted in a reduction of interactions within the middle of the molecule near the mutation. These are accompanied by increases in cross-links with residues in the N- and C-termini now interacting with the disrupted region. Several of the cross-links between the N-terminus and central region spanning residues 140–185 are outside of the original distance constraints for both the consensus monomer and crystal oligomeric structures indicating a major change in the natural folding pattern of APOA1. While we cannot differentiate whether our cross-links are intra- and intermolecular, we note that the overlay on the crystal inter-molecular maps shows a major decrease in the cluster of cross-links in the central region that appears critical for intermolecular interaction in both structures. Overall, our findings suggest that the structural disruption in APOA1^K107del^ in the regions spanning 80 and 140 may lead to more exposure that allow for binding interactions with N- and C-termini in these regions.

## Discussion

**Fig. 5:**
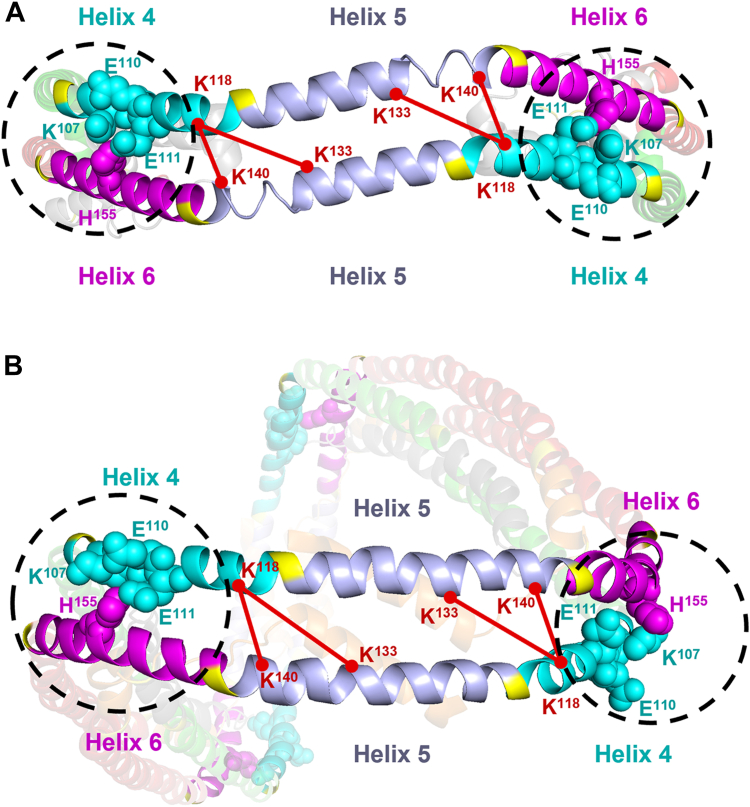
Structural mapping of differential cross-links and saline network involving K^107^ in APOA1^1-183^ and APOA1^43-243^ crystallographic models. K^107^ and its associated salt-bridge network (black dashed circle) involving residues E^110^, E^111^, and R^155^, are shown in the context of the helical regions as defined by proline-induced interruptions (in yellow). A: Mei dimeric crystal structure of APOA1^1–183^. B: Borhani tetrameric model crystal structure of APOA1^43-243^.

Using chemical cross-linking, we demonstrated that deletion of K107 significantly disrupts protein folding and hinders lipid-free APOA1’s ability to properly self-assemble. Our findings further reveal that this disruption alters the interactions and dynamics of the N- and C-terminal domains, offering important clues into how K107 deletion potentially impairs HDL formation and function. Two reported x-ray crystal structures, one depicting APOA1 in a dimeric form (APOA1^1-184^) (23) and the other in a tetrameric form (APOA1^44-243^) (35) show self-association of APOA1 relies on a helix-swapping mechanism. In both structures, K107 plays a critical role in stabilizing the domain swap through formation of intramolecular salt bridges between K107, E110 and E111 in Helix 4 with H155 in the Helix 6 of an opposing APOA1 molecule (Fig. 5). Our data indicate that deletion of K107 drastically destabilizes this region by disrupting these critical interactions as first proposed by Gorshkova and colleagues (17).

Several lines of data support a compromised domain swap in APOA1^K107del^. First, we observed consistent shifts in intrinsic fluorescence measurements, indicating increased exposure of tryptophan residues in a not present in the native APOA1 structure. Given the proximity of the neighboring W108 to K107, it is likely that the increase observed is at least partially attributed to increased exposure of W018. Second, our cross-linking analysis revealed a decrease in interactions between K118-K140 and K118-K133, which are located on the edge of Helices 4 and 5 and are proximal to the region involved in K107-dependent intermolecular interactions (Fig. 5). Interestingly, we also observed a significant increase in crosslinks between K96-K106 in APOA1^K107del^, a cross-link that is absent in wild-type APOA1, suggesting a non-native rotation of that helical region. This is further supported by a threefold decrease and virtual absence of the proximal K77–K88 cross-link in APOA1^K107del^ monomers and higher-order oligomers, respectively. All of these altered cross-links fall within a region on APOA1 shown to be critical for dimer formation and necessary for subsequent membrane-binding activity of APOA1 in HDL (36).

Our analysis also revealed increased interactions between the N-terminal domain of APOA1^K107del^ and regions within Helices 4–6. We note that while most of the cross-links we detected in APOA1^K107del^ satisfy the distance constraints of the consensus structure of monomeric APOA1 (19) cross-links in the N- and C-terminal domain are highly abundant. This suggests there is partial preservation of correct folding but that disruptions in stable helical regions lead to altered conformations, wherein the globular N-terminal domain engages in non-specific binding to exposed amino acids in the central domain normally occupied and stabilized by the domain swap. We see these increases in these interactions in the higher-order oligomers as well. Thus, while the disruption of the domain swap explains the shift to an increase in monomeric protein in the APOA1^K107del^, it could be that the non-specific N-terminal interactions in the mutant are responsible for the small amounts of residual dimer, trimer, and tetramer that remain in the mutant. While these misfolded monomers and “aggregates” might be insufficient for triggering amyloidogenicity alone (9, 13, 14), they may act as pathological seeds for fibril formation observed in carriers of the K107 deletion who present with severe atherosclerosis (37, 38, 39, 40, 41). Indeed, studies on the well-characterized APOA1 mutant G26 R (39, 42) specifically implicated the N-terminal 1–83 fragment in promoting fibril formation (34), supporting the notion that similar misfolding events in the K107 mutant underly amyloid formation and associated cardiovascular pathology.

Increased non-specific binding of the N-terminal domain in APOA1^K107del^ observed in our study may also impede its ability to form nascent HDL. The N-terminus of APOA1 exhibits significant conformational flexibility in both lipid-free state (43) and lipid-bound states (44), a trait recently shown to be crucial for solubilizing phospholipids into nascent HDL discs (45), a key step in HDL biogenesis. Typically, HDL formation proceeds through interactions between the C-terminus of APOA1 and lipid at the cell surface. Upon initial lipidation, the N-terminal helix bundle unfurls to accommodate additional phospholipids and cholesterol, forming a disc-shaped nascent HDL particle. While our lipid-binding assay revealed that the K107 deletion had a negligible impact on lipid interactions, it is important to note that we used planar lipids in the assay, which may not reflect the native curvature of lipoprotein particles. Thus, it remains plausible that while C-terminal lipid interactions are preserved, the N-terminal domain’s ability to fully unfold and stabilize nascent HDL formation is compromised. This aligns with Gorshkova and colleagues, who noted that APOA1^K107del^ exhibits impaired formation of larger-sized HDL particles (21). Previous work has shown that the K107del mutant promotes cholesterol efflux via ABCA1 to a similar extent as wild-type APOA1 (12, 15, 46), suggesting that the mutation does not impair this pathway.

In summary, our study reveals that the loss of K107 significantly disrupts the folding mechanism of APOA1, particularly affecting the N-terminal domain, which plays a pivotal roles in lipid-binding and amyloid formation. This disruption appears to result in non-specific binding and diminished flexibility, contributing to the pathologies observed in carriers of the APOA1^K107del^ mutation. These findings highlight the intricate interplay between APOA1 conformational stability and functional dynamics, underscoring the need for deeper structural analysis of APOA1 mutations to understand their impact on lipid metabolism and cardiovascular health.

Limitations of the study: Our recombinantly expressed APOA1 lacks the first two native amino acids, which were omitted from both wild-type APOA1 and APOA1^K107del^ protein constructs. While these modifications appear to have a negligible influence on our funding, an impact on protein cannot be completely ruled out. Additionally, our lipid-binding assay used planar lipids, which may not accurately reflect native lipid surface curvature encountered by APOA1 on native lipoproteins. Future studies employing different-sized lipoproteins with different curvatures may provide further insights into the effects of the K107 deletion on lipid-binding dynamics. Similarly, our Langmuir trough experimental setup does not allow for re-expansion of lipid films, limiting our ability to directly differentiate between exclusion and rearrangement of surface proteins. While our results align with prior studies demonstrating protein exclusion at high lipid surface pressures, and this interpretation remains consistent within the established framework of APOA1-lipid interaction studies, future studies using alternative approaches (e.g., re-expansion-enabled methods) may better delineate these mechanisms. Another limitation involves our cross-linking data, which cannot definitively distinguish between inter- and intra-molecular cross-links. Employing heavy-labeled proteins mixed with non-labeled APOA1 prior to cross-linking could address this issue by precisely mapping interactions and domain swaps critical to proper APOA1 folding and functionality.

## Data availability

The data supporting this study are available from the corresponding authors upon request. The raw mass spectrometry data are openly accessible for download at Mass Spectrometry Interactive Virtual Environment (MassIVE) community (https://doi.org/10.25345/C5T14V25G).

## Supplemental data

This article contains supplemental data.

## Conflict of interests

The authors declare the following financial interests/personal relationships which may be considered as potential competing interests: AG070480, R01 NS125591]; the International Union of Biochemistry and Molecular Biology (IUBMB) [grant number WW2019]; the Consejo Nacional de Investigaciones Científicas y Técnicas (CONICET) [grant numbers PUE 22920160100002, PIP 11220200102381, PIP 0800, Doctoral Fellowship BECA DOC 15]; the Agencia Nacional de Promoción Científica y Tecnológica (ANPCyT) [grant number PICT-201-0613]; and the Universidad Nacional de La Plata (UNLP) [grant numbers M234, PPID M014, Jóvenes Investigadores 100 #1931/2/19].
