# Supplementary material for: The congenital APOA1 K107del mutation disrupts the lipid-free conformation of monomeric APOA1 and impairs oligomerization
Source: J Lipid Res. 2025 Oct 29;66(12):100929. doi: 10.1016/j.jlr.2025.100929 (PMC12686907; doi:10.1016/j.jlr.2025.100929)
Supplement: Supplementary Data 1 [file mmc1.docx]

**SUPPLEMENTAL INFORMATION:**

**The congenital APOA1 K107del mutation disrupts the lipid-free conformation of monomeric APOA1 and impairs oligomerization.**

Ivo Díaz Ludovico^1,2,3^, Marina C. Gonzalez^2,3^, Horacio A. Garda^2,3^, Romina F. Vázquez^2,3^, Sabina Maté^,3^, María A. Tricerri^2,3^, Nahuel A. Ramella^2,3^, Shimpi Bedi^4^, Jamie Morris^4^, Scott E. Street^4^, Esmond Geh^4^, Geremy C Clair^1^, W. Sean Davidson^4^ and John T. Melchior^1,4,5^

^1^Biological Sciences Division, Pacific Northwest National Laboratory, Richland, WA, USA

^2^Instituto de Investigaciones Bioquímicas de La Plata “Prof. Dr. Rodolfo R. Brenner” (INIBIOLP), CONICET, CCT-La Plata, La Plata, Buenos Aires, Argentina, CP 1900

^3^Facultad de Ciencias Médicas, Universidad Nacional de La Plata (UNLP), La Plata, Buenos Aires, Argentina, CP 1900

^4^Department of Pathology and Laboratory Medicine, University of Cincinnati College of Medicine, 2180 E Galbraith Road, Cincinnati, 45237-0507, USA.

^5^Department of Neurology, Oregon Health and Science University, Portland, Oregon 97239 USA


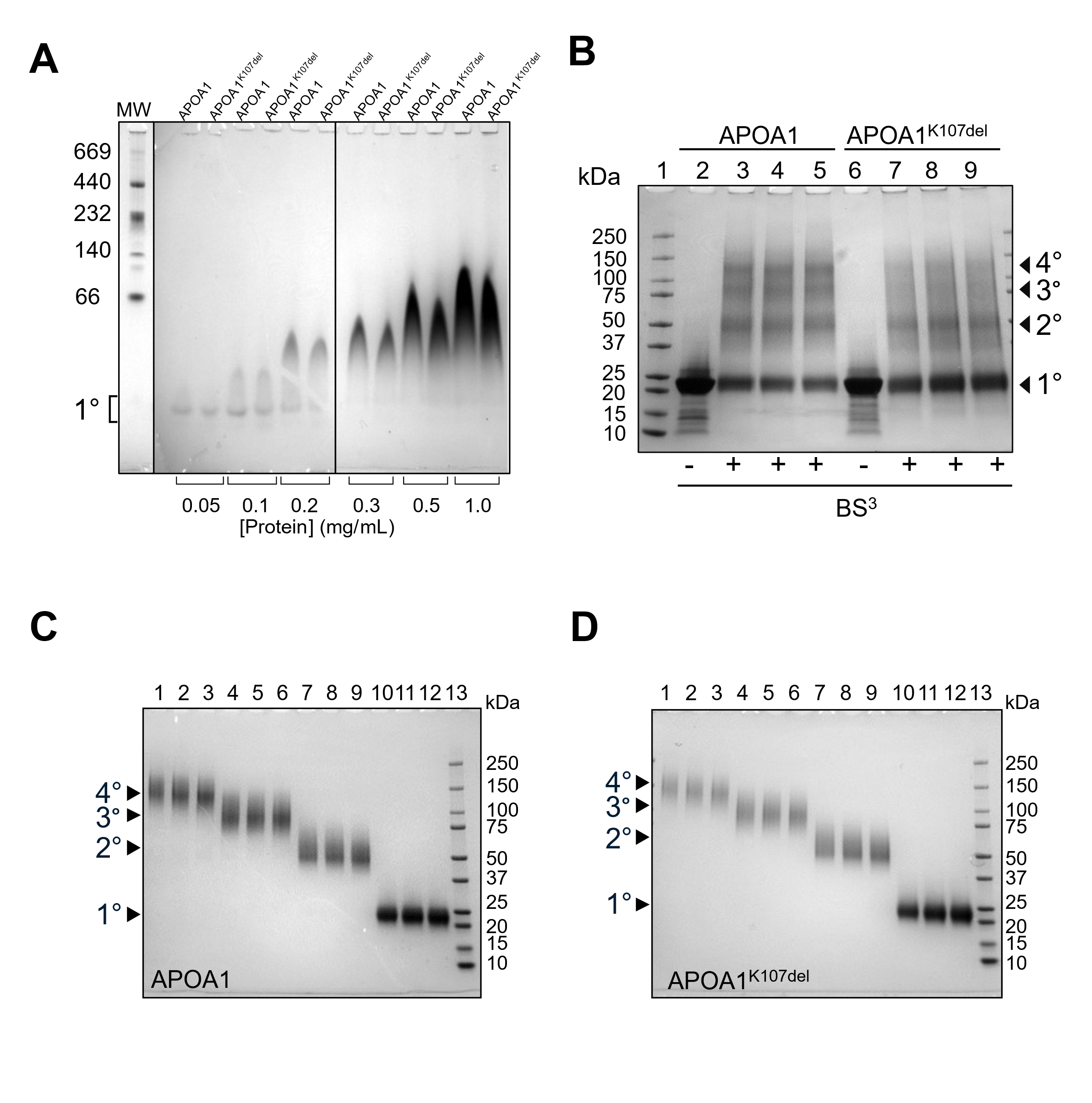


**Supplemental Figure 1. Self-association of APOA1 and APOA1^K107del^ and purity of isolated oligomers.** A) 4-15% Native-PAGE gradient gel of APOA1 and APOA1^K107del^ at different concentrations. Equal volumes of proteins were loaded and visualized by silver stain. B) Proteins were crosslinked with BS^3^ and analyzed by 4-15% SDS-PAGE gradient gel. Each lane contains 8 µg of protein visualized by staining with Coomassie blue. Lane 1: molecular weight marker, Lanes 2 and 6: Proteins with no BS^3^, Lanes 3-5 and 7-9: crosslinked replicates of APOA1 and APOA1^K107del^, respectively. Crosslinked oligomers were isolated by size-exclusion chromatography and purity of APOA1 (panel C) and APOA1^K107del^ (panel D) species were evaluated by SDS-PAGE 4-15% gradient gel. Proteins were visualized by staining with Coomassie blue. Lanes 1-3: tetramer, Lanes 4-6: trimer, Lanes 7-9: dimer, Lanes 10-12: monomer, Lane 13: molecular weight marker.


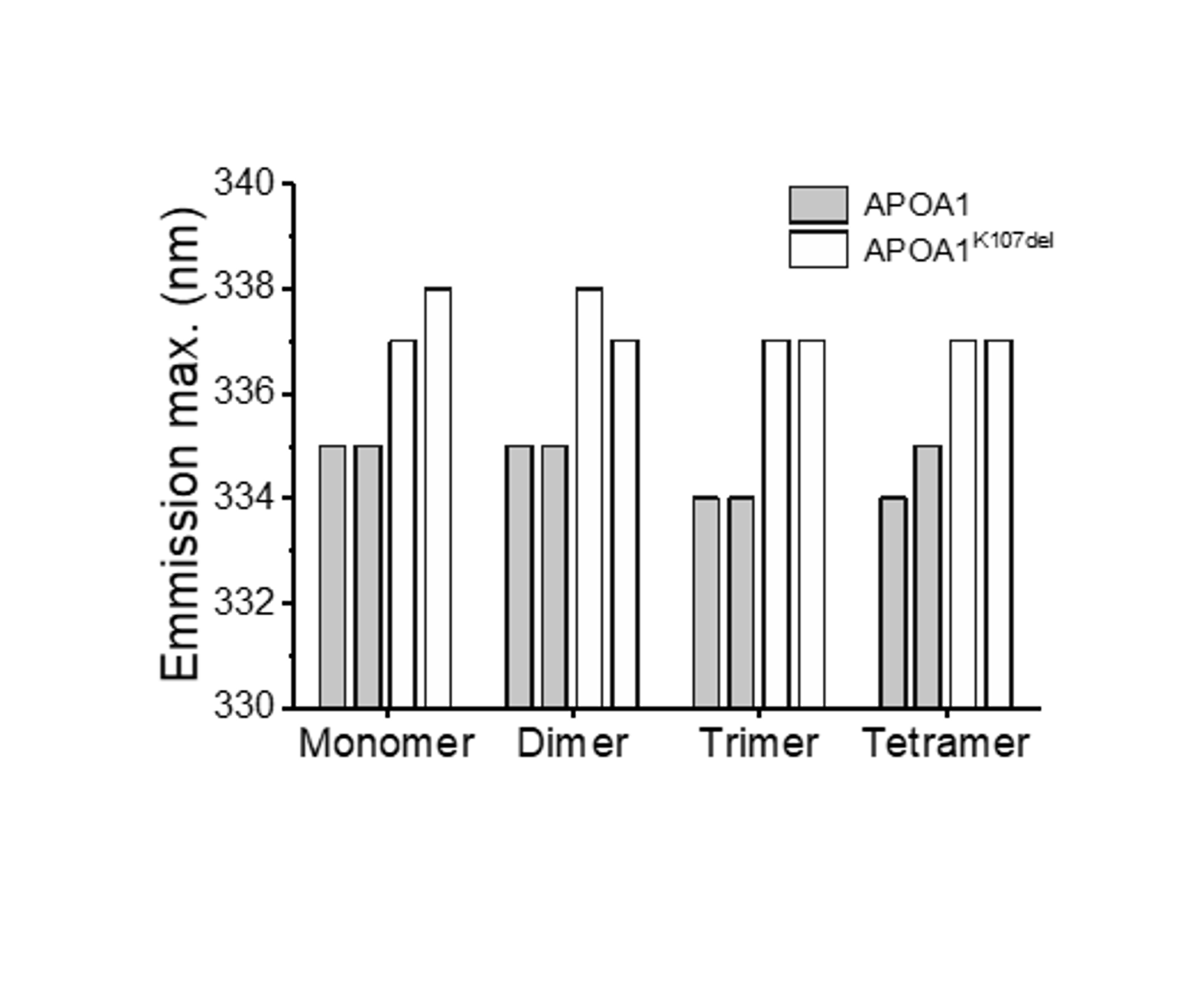


**Supplemental Figure 2: Intrinsic tryptophan fluorescence of cross-linked APOA1 and APOA1^K107del^.** Emission spectra maximum from two independent experiments of each crosslinked oligomer after their isolation by size-exclusion chromatography.
